# A Child With Cleidocranial Dysplasia Presenting With Seizure Disorder

**DOI:** 10.7759/cureus.89765

**Published:** 2025-08-10

**Authors:** Sudipta Mohakud, Amit Satapathy, Subhakanta Patel, Pradosh Kumar Sarangi, Pavithra S, Dhirendra Mohanty

**Affiliations:** 1 Radiodiagnosis, All India Institute of Medical Sciences, Bhubaneswar, Bhubaneswar, IND; 2 Pediatrics, All India Institute of Medical Sciences, Bhubaneswar, Bhubaneswar, IND; 3 Radiodiagnosis, All India Institute of Medical Sciences, Deoghar, Deoghar, IND

**Keywords:** autosomal dominant disorder, cleidocranial dysplasia, seizure disorder, skeletal dysplasia, wormian bones

## Abstract

Cleidocranial dysplasia (CCD) is a rare autosomal dominant disorder of membranous bones with characteristic radio-morphological manifestations. There is disordered ossification of the skull vault and the clavicle, leading to a wide-open anterior fontanelle, frontal bossing, persistent metopic suture, multiple wormian bones, and hypermobile shoulders. Other features are absent nasal bones, the presence of supernumerary teeth, absent ossification of the pubic rami causing pseudo-widening of the pubic symphysis, pseudo-epiphyses of metacarpals and metatarsals, short distal phalanges, and a bell-shaped thorax. CCD is a form of skeletal dysplasia that goes undetected until it presents with some of the associated complications or other diseases. We report the case of a seven-year-old girl presenting with seizure disorder to us, and a detailed clinico-radiological study showed CCD.

## Introduction

Cleidocranial dysplasia (CCD), also known as Marie-Sainton disease, is an autosomal dominant heritable disorder with variable expressivity characterized by retarded membranous bone ossification [1]. The most commonly affected bones are the calvarial bones and the clavicles. It is a rare disorder with a reported incidence of one in a million. Very few cases of CCD associated with neurological disorders have been reported in the literature [2]. We are reporting a case of CCD presenting with a seizure disorder.

## Case presentation

A seven-year-old girl, first in birth order, born to nonconsanguineous parents, was brought to the pediatric outpatient department for episodes of abnormal body movements for about two days, associated with transient loss of consciousness. There was no neurological deficit. There was no history of loss of bladder or bowel control during these episodes. There was no history of fever, fall from a height, headache, nausea, or vomiting. There was no history of birth asphyxia or developmental delay. There was no family history of seizures. The child was vaccinated as per the Universal Immunization Program (UIP) schedule. The child was admitted for evaluation, given a possible seizure disorder.

**Figure 1:**
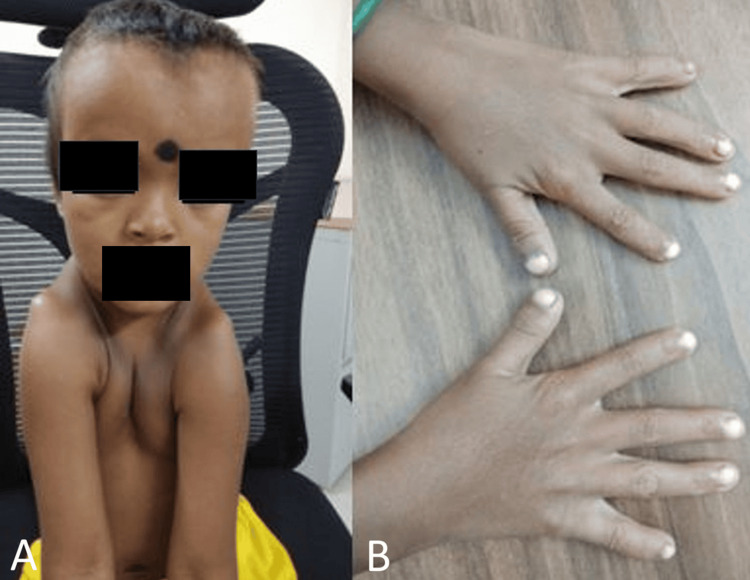
A) Clinical photograph showing characteristic craniofacial features, including flattened nasal bridge, hypertelorism (widely spaced eyes), frontal bossing, and shoulders positioned anterior to the chest. (B) Both hands demonstrating short, stubby fingers.

On examination, there was some syndromic facies showing a flattened nasal bridge with widely spaced eyes (hypertelorism), frontal bossing, and a wide-open anterior fontanelle. The patient could approximate the shoulders in front of the chest. Fingers were short and stubby (Figure 1). Systemic examination was noncontributory. Fundus examination was normal. The child had a weight of 14 kg (<3rd percentile as per the Indian Academy of Pediatrics growth chart) and a height of 99 cm (<3rd percentile as per the Indian Academy of Pediatrics growth chart).

**Figure 2:**
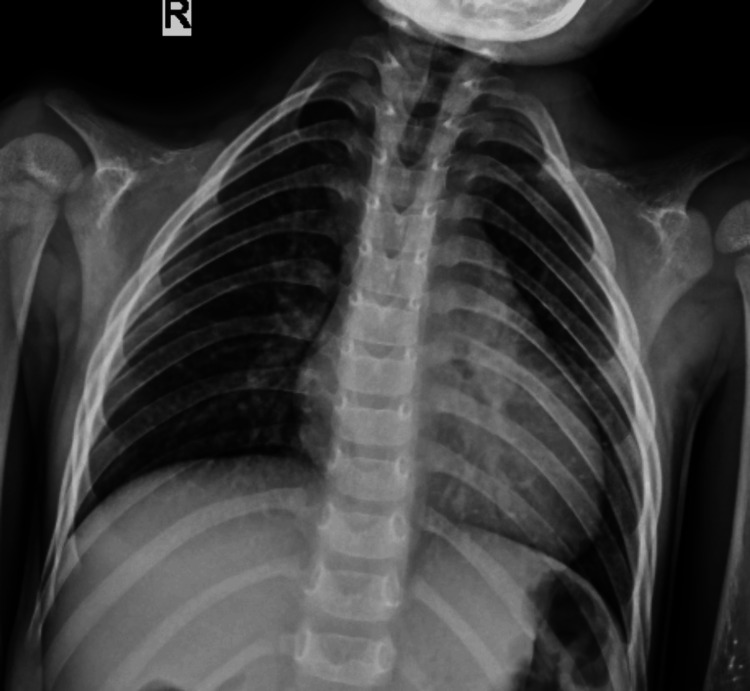
Frontal chest radiograph of this seven-year-old girl showing bilateral absence of clavicles and a bell-shaped thoracic cage.

**Figure 3:**
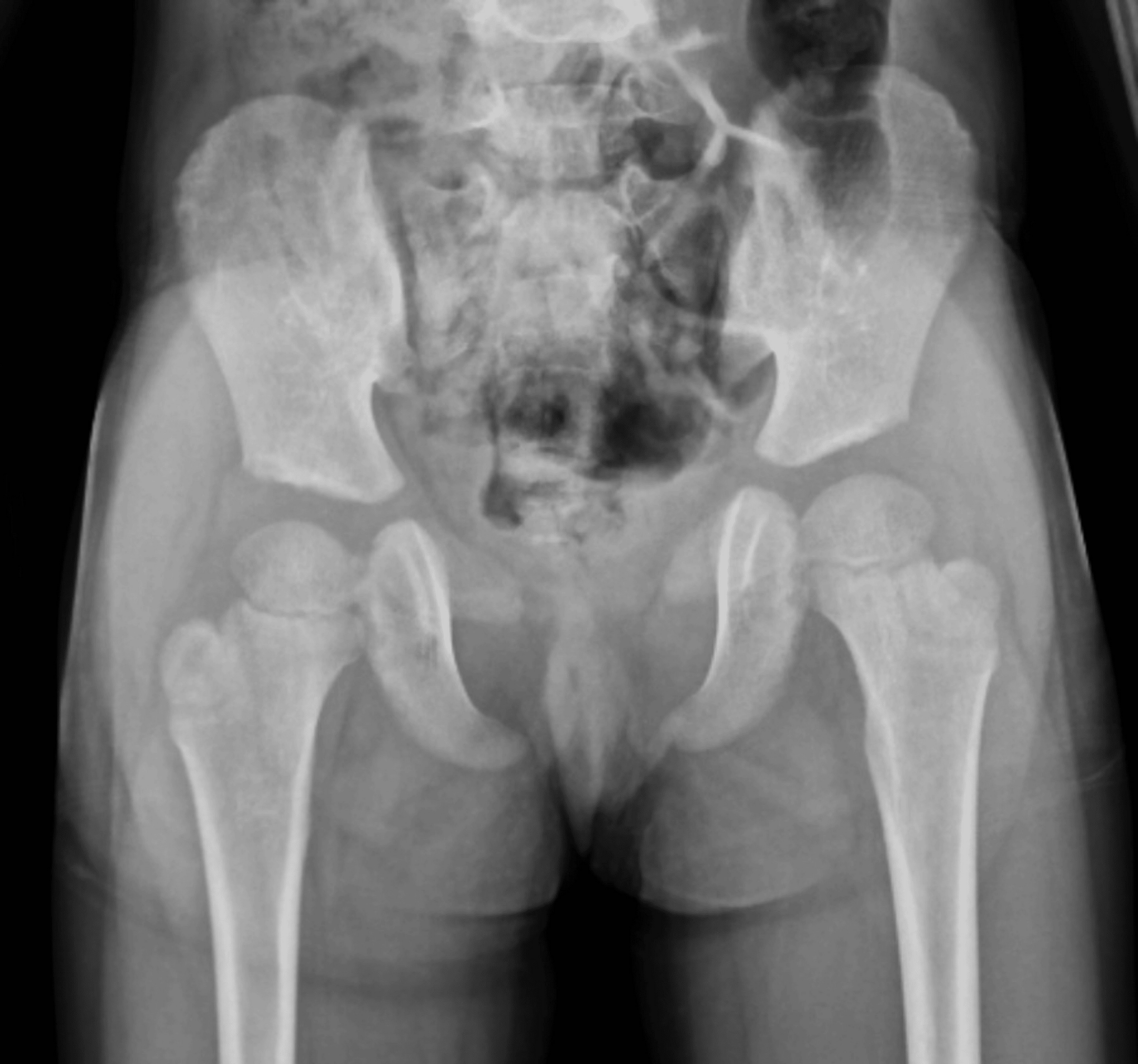
Frontal pelvic radiograph showing bilateral absence of the pubic bones with pseudo-widening of the pubic symphysis.

**Figure 4:**
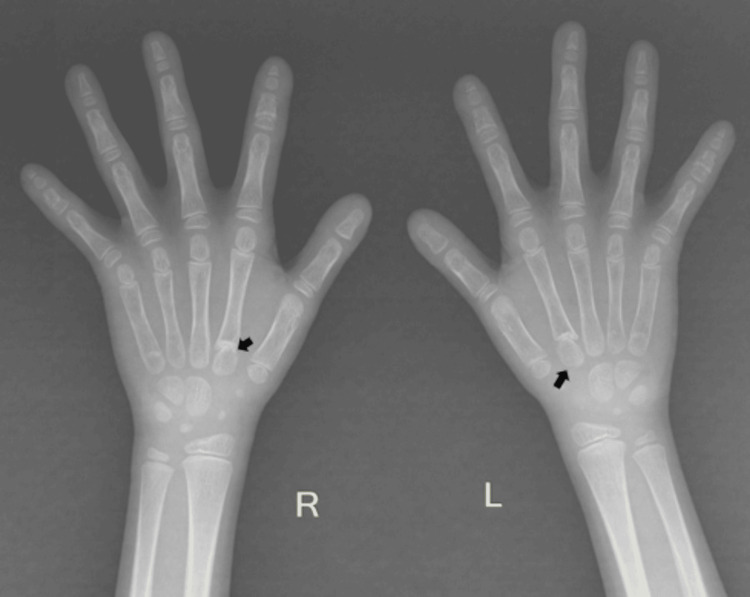
Radiograph of both hands of this seven-year-old girl showing short and pointed distal phalanges. Pseudoepiphyses (black arrows) are noted at the base of the metacarpals of both index fingers.

**Figure 5:**
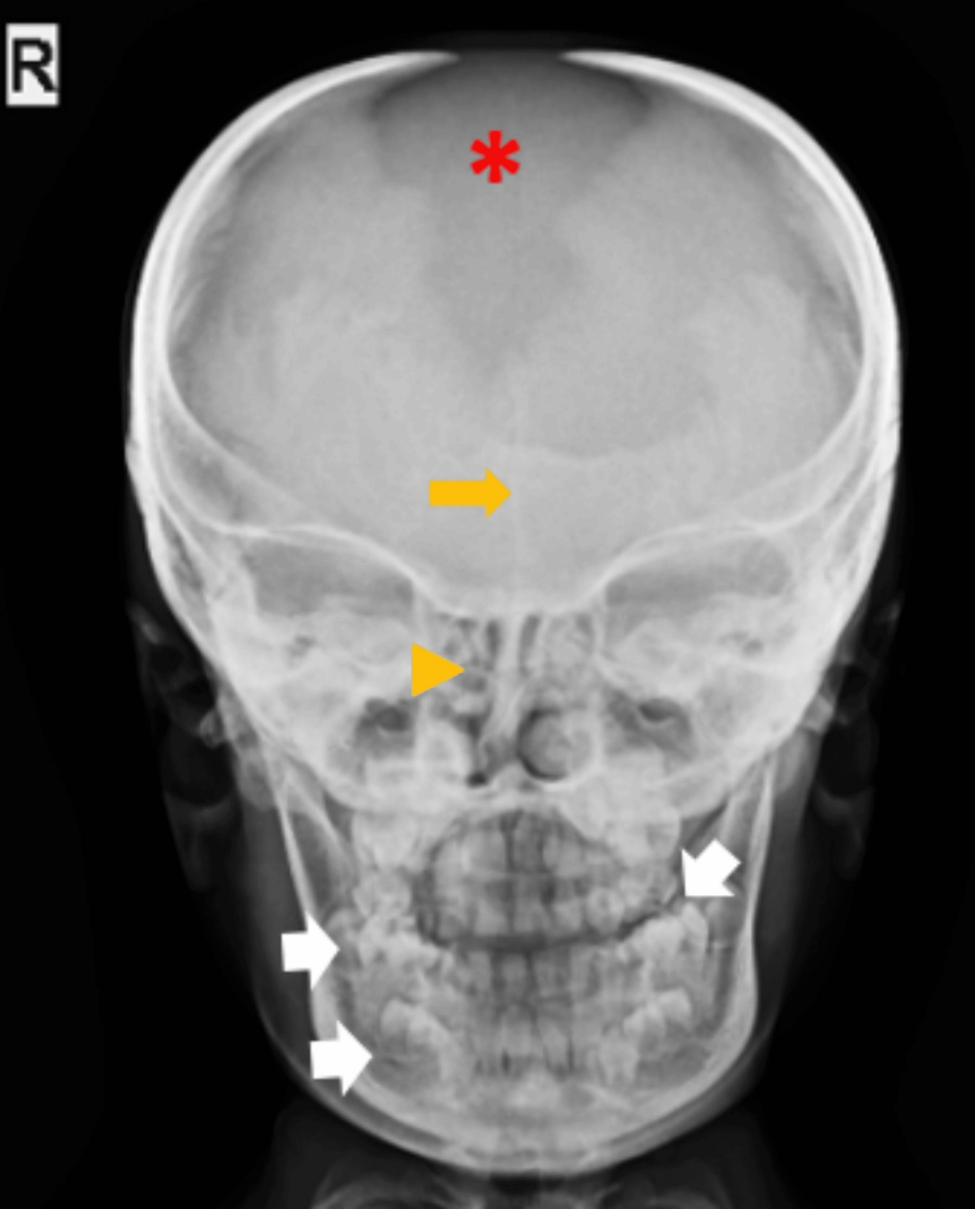
Frontal skull radiograph demonstrating a persistent metopic suture (arrow), absent nasal bones (arrowhead), a wide-open anterior fontanelle (red asterisk), and multiple supernumerary teeth (white arrows).

**Figure 6:**
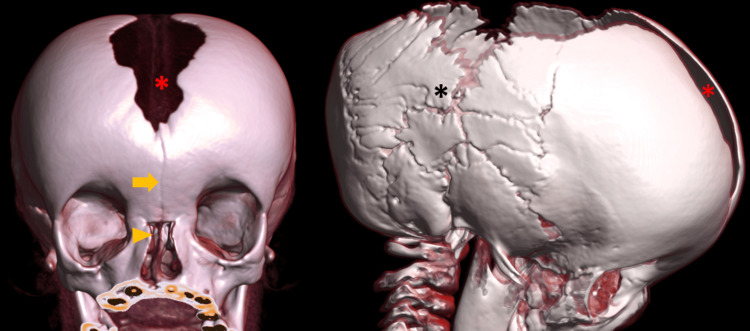
Volume-rendered CT images of the skull showing multiple wormian bones (black asterisk), persistent metopic suture (arrow), frontal bossing, absent nasal bones (arrowhead), and a wide-open sagittal suture and anterior fontanelle (red asterisk).

Given short stature with dysmorphism, the possibility of skeletal dysplasia was considered, for which a skeletal survey was done. Blood investigations were also performed to rule out metabolic bone disorders closely mimicking various skeletal dysplasias. The laboratory findings, namely, complete blood count, renal function tests, serum calcium and phosphorus levels, liver function tests, and thyroid function tests, were within normal limits. The echocardiography was normal and was performed to rule out any congenital cardiac defect. The ultrasound of the abdomen was normal and was done mainly to evaluate renal status. Initially, a computed tomography (CT) scan of the brain was performed to look for the cause of the seizure because of its ready availability and low cost, which showed no abnormality. Then an MRI of the brain and pituitary gland was normal, and it was done to rule out a pituitary cause of short stature. The EEG showed epileptic activity. On a skeletal survey, the frontal chest radiograph showed bilaterally absent clavicles and a bell-shaped thorax (Figure 2). The frontal radiograph of the pelvis showed absent bilateral pubic bones with pseudo-widening of the pubic symphysis (Figure 3). X-ray of the hands showed short and pointed distal phalanges. A pseudoepiphysis (black arrows) was seen at the base of the metacarpals of both index fingers (Figure 4). The frontal radiograph of the skull (Figure 5) and volume-rendered CT images of the skull (Figure 6) showed multiple wormian bones (black asterisk), persistent metopic suture (arrow), frontal bossing, absent nasal bones (arrowhead), and a wide-open sagittal suture and anterior fontanelle (red asterisk). The skull X-ray also showed multiple supernumerary teeth (white arrows). The oral cavity was also examined. All the primary teeth had erupted, and she had not yet lost a baby tooth. A detailed family history was obtained. No similar condition was seen in the parents, siblings, or other family members. Based on the above radiological findings, the child was diagnosed with cleidocranial dysostosis. Genetic testing was offered, but the parents declined because of poor affordability.

The differential diagnosis includes Yunis-Varon syndrome, hypothyroidism, rickets, and hypophosphatasia. Yunis-Varon syndrome also presents with clavicular aplasia, but it is associated with intellectual disability and various other skeletal anomalies [1]. Hypothyroidism presents with multiple wormian bones, short stature, and abnormal thyroid function tests. Rickets and hypophosphatasia present with delayed closure of fontanelles and frontal bossing, along with abnormal serum calcium and phosphorus levels [1,2].

She was given syrup valproic acid at a dose of 20 mg/kg/day. In the follow-up visit after one month, the patient was doing well, and the seizure was controlled. There have been no further episodes of seizure after starting sodium valproate to date.

## Discussion

CCD is inherited or acquired due to a spontaneous mutation. The gene responsible for CCD is RUNX2, located on chromosome 6p21. Only about 70% of patients are found to have genetic abnormalities involving the RUNX2 gene. RUNX2 is responsible for osteoblast differentiation and intramembranous as well as endochondral ossification [3]. The condition may be detected at any age, and there is no specific gender predilection. Individuals usually present with a wide variety of craniofacial bony abnormalities, supernumerary teeth, clavicular aplasia or hypoplasia, and short stature [3]. Classically, the skull vault, not the base, is affected. There is a wide open anterior fontanelle due to non-ossification of the calvarial bones and persistent metopic suture, causing frontal bossing. Multiple intrasutural bones (wormian bones) are seen mostly in the lambdoid suture. Early fusion of the coronal suture causes brachycephaly. The maxilla may be hypoplastic. Delayed fusion of the symphysis menti and supernumerary teeth with dentition difficulties is seen. Absent or small nasal bones give the appearance of a flattened nasal bridge and hypertelorism. Unilateral or bilateral hypoplastic or aplastic clavicles are characteristic findings. Scapular development is also affected. These abnormalities cause excessive mobility of the shoulder girdles. The thorax appears bell-shaped. Supernumerary ribs may be seen. The pelvis is narrow, and the absence of pubic bone ossification causes pseudowidening of the pubic symphysis. The distal phalanges are hypoplastic, with cone-shaped epiphyses of the middle phalanx giving a short, thick appearance to the fingers. Pseudoepiphyses of metacarpals and metatarsals are seen more characteristically at the base of the second metacarpal [1,4-8]. Normal intelligence is the rule. Associations with epilepsy or other seizure disorders, as well as syringomyelia, have been reported in the literature and might suggest conditions with chromosomal abnormalities involving additional genes beyond RUNX2 [9-11]. CCD predisposes patients to various morbidities; however, these individuals generally have a normal life expectancy [8].

Thus, our case is a sporadic form of CCD. Whether the occurrence of seizures is unrelated to CCD or has some pathophysiological association with the disease could not be clarified, and it presents a new research question.

## Conclusions

CCD, though a rare congenital disorder, should be considered in the differential diagnosis of children presenting with a wide-open anterior fontanelle. The hallmark radiological findings - such as absent or hypoplastic clavicles with hypermobile shoulders - aid in early identification. Although present from birth, the condition is frequently diagnosed later in childhood or adulthood due to short stature, delayed dentition, or craniofacial abnormalities. Early recognition is essential to prevent complications such as malocclusion and facial dysmorphism. Radiologists and clinicians must be familiar with the diverse radiomorphological features of CCD to facilitate timely diagnosis and intervention. Genetic counseling for parents and screening of siblings are recommended to enable early detection and appropriate management.

## References

[1] Minocha P, Choudhary A, Sitaraman S (2017). Cleidocranial dysplasia: a rare case report. J Med Sci.

[2] Ma Y, Zhao F, Yu D (2019). Cleidocranial dysplasia syndrome with epilepsy: a case report. BMC Pediatr.

[3] Yoshida T, Kanegane H, Osato M, Yanagida M, Miyawaki T, Ito Y, Shigesada K (2002). Functional analysis of RUNX2 mutations in Japanese patients with cleidocranial dysplasia demonstrates novel genotype-phenotype correlations. Am J Hum Genet.

[4] Sivan MP, Jayakumar K, Jayalakshmi PS, Sruthi N (2017). Classic case of cleidocranial dysplasia with an infected mandibular cyst. Int J Case Rep Images.

[5] Mohan RP, Suma GN, Vashishth S, Goel S (2010). Cleidocranial dysplasia: clinico-radiological illustration of a rare case. J Oral Sci.

[6] Medina O, Muñoz N, Moneriz C (2017). [Cleidocranial dysplasia: a case report]. Rev Chil Pediatr.

[7] Singh S, Sharma S, Singh H, Wazir ND (2014). Cleidocranial dysplasia: a case report illustrating diagnostic clinical and radiological findings. J Clin Diagn Res.

[8] Dixit R, Dixit K, Paramez AR (2010). Cleidocranial dysplasia. Lung India.

[9] Engel J Jr (2013). Seizures and Epilepsy, 2nd Edition.

[10] Back SJ, Pollock AN (2013). Cleidocranial dysostosis. Pediatr Emerg Care.

[11] Oyer CE, Tatevosyants NG, Cortez SC, Hornstein A, Wallach M (1998). Cleidocranial dysplasia with neonatal death due to central nervous system injury in utero: case report and literature review. Pediatr Dev Pathol.

